# Multi-country SDGs indicator projections involving impacts of economic shocks: A protocol applied to the COVID-19 pandemic for ASEAN-5 countries

**DOI:** 10.1016/j.mex.2022.101772

**Published:** 2022-06-23

**Authors:** Ahmad Komarulzaman, Zuzy Anna, Arief Anshory Yusuf, Venkatachalam Anbumozhi

**Affiliations:** a Center for Sustainable Development Goals Studies, Padjadjaran University, Jl. Dipati Ukur No. 46, Bandung, Indonesia; b Economic Research Institute for ASEAN and East Asia (ERIA), Sentral Senayan ll, 6th Floor, JL. Asia Afrika, No. 8, Gelora Bung Karno, Senayan, Jakarta, Indonesia

**Keywords:** Sustainable development goals, UN Agenda 2030, Forecast, Counterfactual projection

## Abstract

Economies often experience large shocks, necessitating the revision of development indicator forecasts, including Sustainable Development Goals (SDGs) indicators. Many of those, predicted for 2030, require continued monitoring and re-estimation of how great the impact of these shocks will be, e.g., comparing the achievements with and without the shocks (counterfactual). In this paper, we design a protocol to create datasets containing 2030 SDGs indicator projection estimates that can be used to monitor the extent to which current economic shocks will affect the trajectories of those indicators. We combine official United Nations Statistics Division (UNSTAT) SDGs indicator data and economic growth projections data and fit them into the protocol. The protocol includes filtering UNSTAT SDGs indicators for regression analysis connecting them with economic growth. We assume that the difference in economic growth projections before and after a shock is primarily caused by the shock. This implies that our protocol is less suitable for an episode of more subtle shocks or shocks with multiple causes. We use these estimates to create the SDGs indicators projection dataset. We applied this to ASEAN-5 countries and the COVID-19 pandemic. The same protocol can be used for other countries as well as other economic shocks.

• The protocol is useful to monitor how previous projection trajectories of SDGs indicators are affected by relevant large economic shocks, such as those due to the COVID-19 pandemic. The resulted dataset can also be used for comparing achievements, with and without shocks (counterfactual).
• This protocol can be used by national and international agencies, especially those in charge of planning, monitoring, and evaluating the SDGs agenda. The protocol and the resulting data would also be helpful to researchers working on SDGs issues.
• In this paper, the protocol to create the projection dataset of SDGs applies for the ASEAN-5 countries using the COVID-19 shocks. These can also be applied for other countries and other economic shocks.

## Graphical abstract

**Figure:**
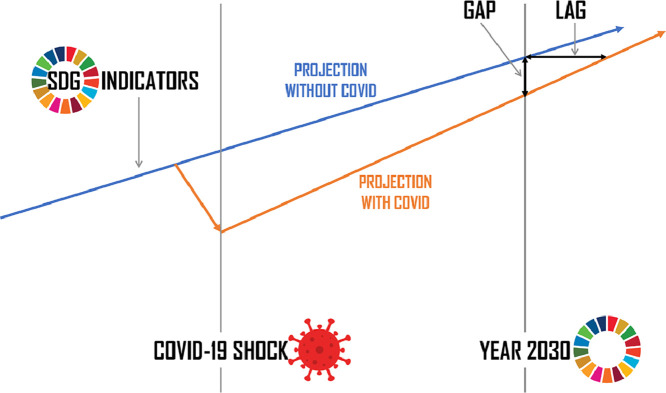

**Specifications table**

**Table:** 

Subject area:	Economics and Finance
More specific subject area:	*Economic Development and Growth, SDGs, Sustainability*
Name of your method:	*Counterfactual SDGs Indicator Projection*
Name and reference of original method:	*[1]**A. Komarulzaman, Z. Anna, A.A. Yusuf, V. Anbumozhi, The Impact of Global COVID-19 Crisis on SDGs Achievement in ASEAN-Countries, in: V. Anbumozhi, K. Kalirajan, F. Kimura (Eds.), Sustainable Development Goals and Pandemic Planning, Springer, Singapore, 2022*. https://doi.org/10.1007/978-981-16-6734-3_2.
Resource availability:	*SDGs UNSTAT dataset:*https://unstats.un.org/sdgs/report/2020/#foreword, *Economic projection data from the IMF economic outlook:*https://www.imf.org/en/Publications/WEO/weo-database/2019/October/download-entire-database, *Economic projection data from the World Bank global:*https://openknowledge.worldbank.org/handle/10986/34710*GNI per capita (constant 2010 US$) and population 2014-2019 from the World Bank Open Data databases:*https://data.worldbank.org/*Population projections data from the EconMap database, version 2.4:*http://www.cepii.fr/CEPII/en/bdd_modele/download.asp?id=11

## Introduction

There is no doubt the coronavirus disease 2019 (COVID-19) pandemic will disrupt the long-term global development plans laid out in the Sustainable Development Goals (SDGs) by the United Nations (UN). Some experts believe that without any shocks, such as the COVID-19 pandemic, the target of achieving the SDGs in 2030 is still tentative, too ambitious, utopian, and, perhaps, impossible [2], [3], [4], [5]. The COVID-19 pandemic has worsened the possibility of achieving the SDGs. Some studies have reported [6,7] that the progress toward achieving the SDGs was slow even before the COVID-19 pandemic, and the pandemic has made it even more difficult.

The extent of the disruption to the SDGs due to COVID-19 is very relevant to ASEAN countries. ASEAN countries agreed to the 2025 vision [8], which principally shares the same agenda as the SDGs [9]. The SDGs are widely shared by ASEAN countries; therefore, the interruption caused by the pandemic needs to be addressed. From an academic perspective, the disruption to achieving SDGs in ASEAN countries is of high relevance. Although ASEAN countries respond to the COVID-19 pandemic quite differently, Vietnam's decisiveness in handling the pandemic and avoiding serious crises is remarkable. Conversely, the Philippines seems to be struggling to contain the virus. Indonesia used a different approach: trying to subtly balance health and economic aspects but with the risk of a prolonged pandemic. These approaches may have different impacts on how the pandemic will affect these countries’ economic development trajectory, including their approach to SDGs.

This study is motivated by the lack of specific analytical research about the impact of COVID-19 on achieving the SDGs and aims to analyze it. From the literature review, we find that some existing research focuses more on the impacts on macroeconomic factors, a qualitative estimation of the socio-economic impacts based on expert views, and quantitative research on one or two economic indicators, such as poverty rates and economic growth.

The UN [10] and United Nations Department of Economic and Social Affairs (UNSDG) [7] published reports on the impact of the pandemic on the SDGs and the global socio-economy; both reports were qualitative analyses. The UN's report [10], which analyzed the implication of COVID-19 for the 17 SDGs, stated that the COVID-19 pandemic has disrupted the agenda of almost all SDGs. Similarly, UNSDG [7] stated that almost all SDGs would be negatively impacted by the COVID-19 pandemic, except for climate action (Goal 13) and inequality (Goal 10). Quantitative estimation of the impact of the COVID-19 pandemic was carried out by Vos et al. [11,12], using the computable general equilibrium (CGE) complex model and data from approximately 30 household surveys mainly from Sub-Saharan Africa and South Asia. They found that a percentage point reduction in the global gross domestic product (GDP) would increase poverty (at the lower World Bank poverty line of US$ 1.90 per day), making an additional 14–22 million people poor. Moreover, ILO [13], using the CGE model by McKibbin and Fernando [14], revealed that due to the pandemic, about 9–35 million workers (at the higher World Bank poverty line of US$ 3.20 per day) became poor in many countries in 2020. IGES [15] reported that the environmental pillars of the SDGs would be of global focus, as there would be short-term issues related to medical waste, air pollution, and unsustainable lifestyles. However, in the long term, there would be issues related to sustainable cities and climate adaptation planning [15]. This view is supported by *The Economist* [16], which reported the concern about a healthy ocean, especially on the escalation of plastic waste in several water bodies globally.

Numerous other studies have discussed the impact of the COVID-19 pandemic on various aspects of development. Regarding those about economic impacts, early studies, especially those during the beginning of the pandemic, mostly used economy-wide modeling [14,^17^,18]. These studies focused on how the pandemic will affect standard macroeconomic variables, such as economic growth or consumption. Broader dimensions of economic development, such as social indicators (e.g., poverty and education) and health were not part of the focus of such economic modeling exercises.

Soon after the pandemic took its course, various analyses on how it may affect the SDGs were published. It seems that the focus was on extreme poverty—SDG 1. The study of Hoy and Sumner [19] is among the first few studies to question the achievability of the UN's SDGs to end extreme poverty in the aftermath of the COVID-19 pandemic. Various international organizations, especially the UN's organizations, also published reports analyzing how COVID-19 would affect the SDGs agenda. For instance, the UNDP [20] found that the social and economic impacts of COVID-19 would widen the gap between people living in rich and poor countries. Furthermore, ILO [21] predicted that the COVID-19 pandemic would disrupt the declining trend of the proportion of the working poor. In its flagship publication of the Sustainable Development Report, the UN [22] concluded that decades of progress in reducing extreme poverty have been halted or reversed, and both inequalities within and between countries are intensified because of the COVID-19 pandemic.

Most of the literature on the impact of the COVID-19 pandemic on SDGs is global in its focus. However, most policies about almost all the SDGs agenda are within nation-state jurisdictions. The need to re-think and re-design policies in anticipation of the impact of and recovery from the COVID-19 pandemic from the perspective of national policymakers is more urgent. This paper aims to aid national policymakers to assess the extent of the challenges faced in achieving the 2030 SDGs. Due to the multi-dimensionality of SDGs and their extensive list of indicators, such an assessment is not a trivial task. Based on quantitative analysis, our proposed protocol can offer insight into how to filter SDGs indicators, considering the impact of the pandemic.

Unlike other studies that have assessed the impact of the COVID-19 pandemic on SDGs, this paper focuses on methodology. In particular, we propose a protocol to estimate an SDGs indicator projection that can be used to monitor the extent to which current economic shocks would affect the trajectories of this indicator from databases common to all countries (i.e., SDGs UNSTATS database). By demonstrating how this protocol works for ASEAN countries, this study reveals that this approach is directly replicable to other countries in the database. However, in this regard (methodology-focused), our study is not the first. Along with other economic variables, some studies [23], [24], [25], [26] have already demonstrated how the pandemic can predict the outcome of various financial indicators, such as stock market returns and exchange rates. We hope that our focus on the impact of the COVID-19 pandemic on SDGs would enrich the existing literature.

## Method

This paper reports with and without COVID-19 pandemic projection of 269 SDGs indicator-series-dimensions among the ASEAN-5 countries, including Indonesia, Malaysia, Philippines, Thailand, and Vietnam. Projection without the COVID-19 pandemic is the SDGs indicator projection for 2030 assuming that the COVID-19 pandemic has abated. This projection is calculated utilizing the 2020–2030 economic growth projection published in 2019 [27]. Meanwhile, the projection with the COVID-19 pandemic is calculated using the 2020–2030 economic growth projection published on January 2021 [28] that has already considered the COVID-19 crisis. These 269 indicator-series-dimensions cover 13 goals, 43 targets, and 55 SDGs indicators (see Table 1).

**Table 1.** Number of Indicator-series-dimension by SDGs

Goals	Number of			
	SDGs Target	SDGs Indicator	Indicator- Series	Indicator- Series- Dimension
1 No Poverty	4	5	18	33
2 Zero Hunger	2	3	4	8
3 Good Health and Well-being	9	14	19	29
4 Quality Education	6	8	14	140
5 Gender Equality	1	1	1	3
6 Clean Water and Sanitation	4	4	4	7
7 Affordable and Clean Energy	1	2	2	4
8 Decent Work and Economic Growth	5	6	7	25
9 Industry, Innovation, and Infrastructure	4	5	6	6
11 Sustainable Cities and Communities	1	1	1	1
12 Responsible Consumption and Production	1	1	1	5
16 Peace, Justice, and Strong Institutions	3	3	3	3
17 Partnerships for the Goals	2	2	3	5
**Total**	**43**	**55**	**83**	**269**

Notes: An indicator-series is the combination of SDGs indicators with their sub-indicators (series). For example, the SDGs indicator “1.1.1 is the proportion of the population living below the international poverty line by sex, age, employment status, and geographic location (urban/rural).” It consists of two series, namely, “Proportion of population below international poverty line (%)” and “Employed population below international poverty line, by sex and age (%)”. These indicator series can have several dimensions. For example, “Employed population below the international poverty line, by sex and age (%)” are disaggregated by sex and age group (15+, 15-24, 25+). In this paper this is called indicator-series-dimension.

**Fig. 1:**
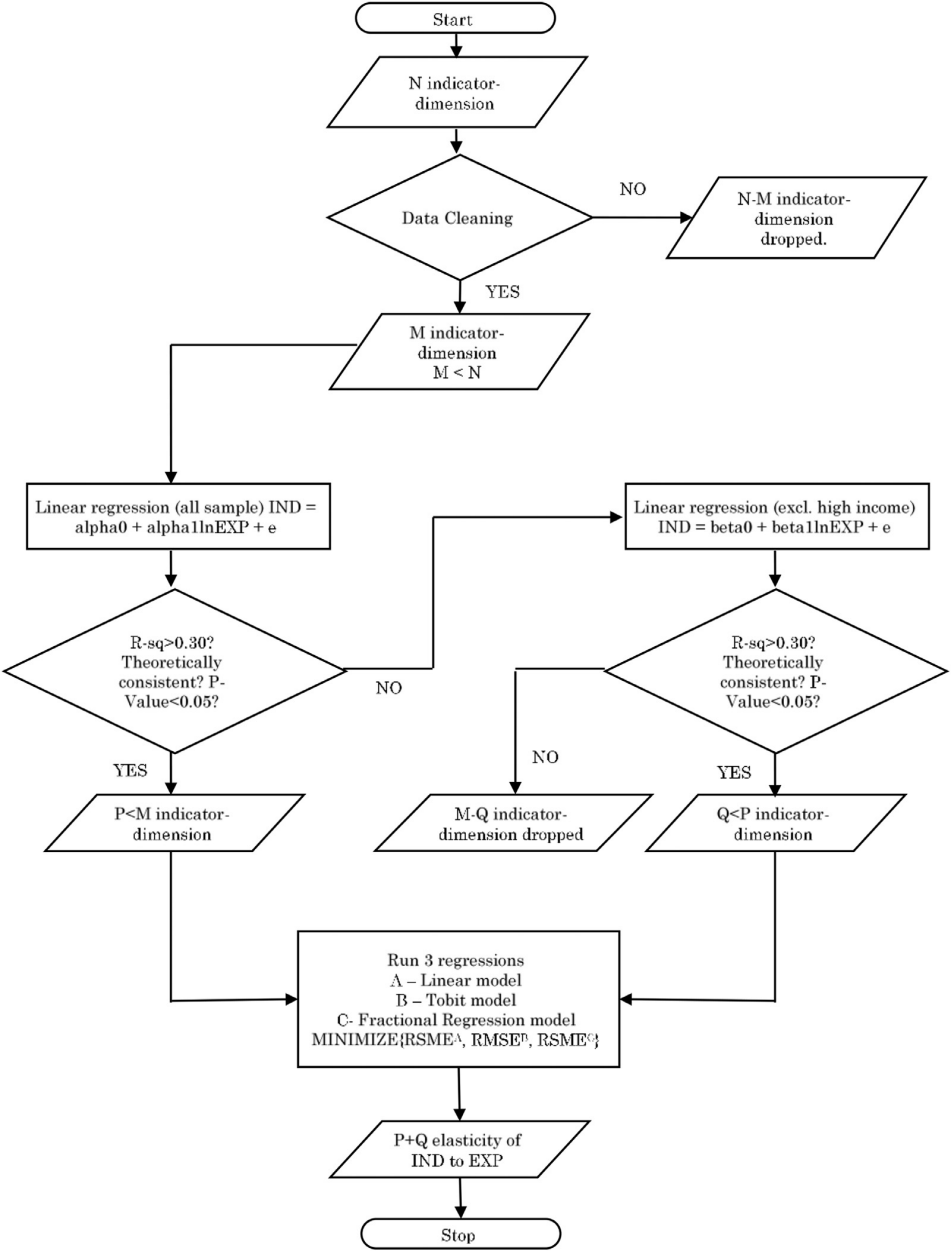
Indicator and model selection protocol. Notes: This figure depicts the selection process of the indicator-series-dimension for the quantitative analysis with and without COVID-19 projections. The indicator-series-dimension (in this figure, shorten to indicator-dimension) is the combination of SDGs indicators with their sub-indicators (series) and dimensions (see notes on Table 1 for an example). N, M, P, and Q are the numbers of the indicator-series-dimension selected in each step. Only those that pass the three criteria (R-square > 0.30, theoretically consistent, and P-value < 5% level) can go through the next process for estimating the elasticity of the SDGs indicator of GNI per capita

The protocol to create with and without the COVID-19 pandemic projection dataset is presented in Fig. 1 and can be described as follows:

1. Data collection
  a. We collected the SGDs data from the UNSTAT database (downloaded August 5, 2020 [29]) and arranged it by 17 SDGs goals, 192 indicators, and 432 series, all with multiple dimensions. The dimensions include sex, age, education level, location, type of Internet speed, type of product, IHR capacity, wealth quantile, type of occupation, activity, disability status, migratory status, and type of skill. Given this combination, we initially identify a unique 1,992 indicator-series-dimension.
  b. The economic projection data with and without the COVID-19 pandemic are gathered from the IMF World Economic Outlook [27] and the World Bank Global Development Prospects [28]. We collected two sets of data—the economic growth projection data that were published just before the COVID-19 pandemic outbreak (for baseline or projection without considering the impact of the COVID-19 pandemic) and the projections published around one year after the pandemic outbreak (projection that considers the impact of the COVID-19 pandemic).
  c. GNI per capita (constant 2010 US$) and population per country for the latest five years (2014-2019) were gathered from the World Bank Open Data databases [30].
  d. Population projections are derived from the EconMap database, version 2.4 [31], to turn GDP growth projections into GNI per capita growth projections. Population growth was subtracted from GDP growth, assuming that the difference between GDP and GNI remains proportionally constant toward the projection period.
2. Data cleaning and filtering

To maintain replicability of the analysis, data cleaning and filtering were conducted as systematically as possible. We closely followed the protocol depicted in Fig. 1. Steps were taken to select indicator-series-dimensions, based on specific criteria from the N = 1,992 to be included in our succeeding analysis.

a. First, we kept indicator-series-dimensions with fairly recent data, i.e., from the last five years (2014–2019). Indicator-series-dimensions were dropped when there were no available data for any of the ASEAN-5 countries. In addition, for each country in the dataset, we make sure that indicator-series-dimension in that particular year has the matching GNI per capita data for the same year. Up to this stage, we have kept M = 732 indicator-series-dimensions to be processed in the next steps.
b. Secondly, a linear cross-country regression analysis of E(y|GNI) = α_0_ + α_1_GNI was run for each of the M indicator-series-dimensions. Considerable information was gathered from this regression analysis, including R-squared, its coefficient of the variable GNI, and the P-value of the coefficient.

Given this information, we performed another selection process using three criteria, namely, R-square > 0.30 [32], a theoretically consistent sign of coefficient, and whether the coefficient is significant with at least a 5% level. Each indicator-series-dimensions have to pass these three criteria to ensure the reliability of the elasticity to be estimated from this cross-country data.

In this process, we used only linear regression, first, because we were able to compare the R-square directly with previous studies using the same method [32], and second, because we found that a non-linear regression model (Tobit or Fractional regression) produces non-comparable R-squared.

However, a non-linear relationship between various SDGs indicators with GNI could be observed, particularly in the domain of high-income countries [32]. Drinking water and sanitation coverage, for example, can be achieved even before reaching very-high-income. Hence, plotting the relationship between GNI per capita and drinking water and sanitation coverage could show as positively sloped in the low- and middle-income domains that are followed with flat curves in the high-income domain. One solution to account for this issue is to drop high-income countries from the analysis [32]. This solution is then applied to re-check the reliability of the relationship of the indicator-series-dimension which did not pass the previous selection process. Similar three selection criteria would then be applied to proceed to the next stage.

At the end of this selection process, there are P+Q = 269 indicator-series-dimensions that can be included in the proceeding analysis.

3. Estimating Elasticity of Income

To project a country's SDGs indicator toward 2030, a reliable or robust relationship between GNI per capita and the SDGs indicators (elasticity) and the projection of the GNI per capita toward 2030 was established. Due to a large number of indicators-series-dimension, this process was done systematically, as follows:

a. First, a cross-country (cross-section) regression between SDGs indicators and GNI per capita is done. It should be noted that in this analysis we included all of the available country data, not limited solely to the ASEAN-5 countries. A pair of country-observations with the most recently available data, that is, 2014–2019 is included. Considering that many of the indicators are proportional and clustered around 100 or zeros around high-income countries, two alternative non-linear regressions are used. In total, there three regression models are used.
i. Linear model, which can be written as:
  (1) $$E\left(y|GNI\right)=\alpha_{0}+\alpha_{1}GNI$$

Where *y* is the observed SDGs indicator, *GNI* is the Gross National Income Per Capita (in constant US$ in logarithmic), *E(y|GNI)* is the expected value of *y* in any given *GNI* and α_0_ and α_1_ are the parameters to be estimated. This model will be estimated using ordinary least square (OLS),

ii. Tobit model, which can be written as:
  (2) $$E\left(y|GNI\right)=\beta_{0}+\beta_{1}GNIandy^{*}=\left\{ \begin{array}{c} \;\;y\;\;\\ \;\;a\;\;\\ \;\;b\;\; \end{array}\begin{array}{c} \text{if}\;\;\\ \text{if}\;\;\\ \text{if}\;\; \end{array}\begin{array}{c} a<y<b\\ y\leq a\\ y\geq b \end{array}\right.$$

Where *y* is the observed SDGs indicator, *y*^⁎^ is a latent (unobserved) variable, and *a* and *b* are the lower and upper thresholds of *y*, respectively. In most cases, *a* = 0 and *b* = 1, that is, the SDGs indicator is normalized into proportion instead of percentage. This model will be estimated using Maximum Likelihood.

iii. Fractional response model, which can be written as:
  (3) $$E\left(y|GNI\right)=\Phi \left(\gamma_{0}+\gamma_{1}GNI\right)$$

Where is the SDGs indicator taking the value between 0 and 1, and is standarized cummulative normal distribution. This model will be estimated using Maximum Likelihood.

b. Second, the regression result with the highest goodness of fit, in this case R-squared or Pseudo-R-squared, are going to the next process. A threshold of value of the (Pseudo-) R-square of 0.30 is used following [32].

**Fig. 2:**
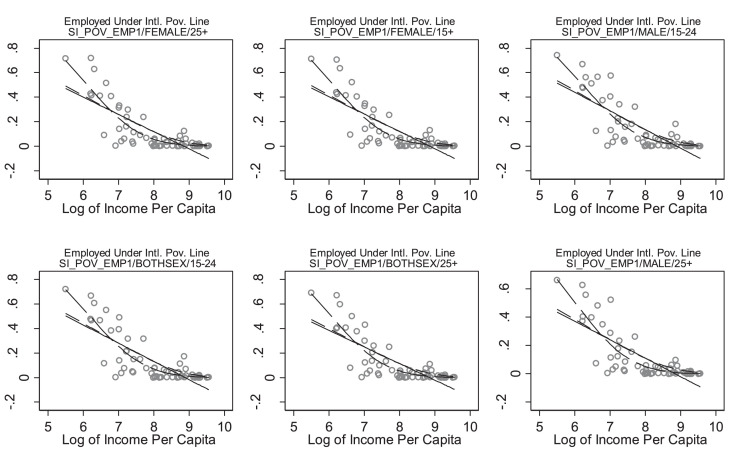
Selected plot of the relationship between indicators of SDGs and income per capita. Notes: These figures plot the log of income per capita with the indicator-series-dimension using the latest available data from the UNSTAT database. The figure is equipped with three fitted lines of three possible regression models: linear, Tobit, and fractional response models

The example of the scatter plot of these indicator-series-dimensions against the log of GNI per capita with all three possible models is depicted in Fig. 2.

c. Third, calculating the elasticity of the SDGs indicator to GNI per capita. We calculate the elasticity for all possible value of GNI per capita that is relevant for the projection due to the non-linearity of the relationship.

4. Projecting SDGs indicator toward 2030 for ASEAN-5 countries.

We use Eqs. 1,2, and 3 to estimate the projected value of SDGs indicators in 2030. For each SDGs indicator, we estimate two sets of data: the baseline projection (the GNI per capita is calculated using the baseline economic growth forecast) and the COVID-19 impact projection (the GNI per capita is calculated using the economic growth forecast that considers the impact of the COVID-19 pandemic).

We assume that the difference in economic growth projection conducted before and after a shock is primarily caused by the shock. Thus, forecasters revise the projection after the shocks. However, this implies that our protocol is less suitable for an episode of more subtle shocks or shocks with multiple causes.

5. Calculate the projection gap and lag.

a. Projection gap is the difference between the with and without COVID-19 projections that are measured in percentage.
b. Projection lag is the number of years delay between the with and without COVID-19 projection.

This paper is also equipped with the calculated impact of COVID-19 on each SDGs indicator-series-dimension in the form of a projection lag-and–gap. Projection gap is the percentage of difference between with and without COVID-19 projections. The projection (time) lag is the number of years delay between the with and without COVID-19 projection.

**Fig. 3:**
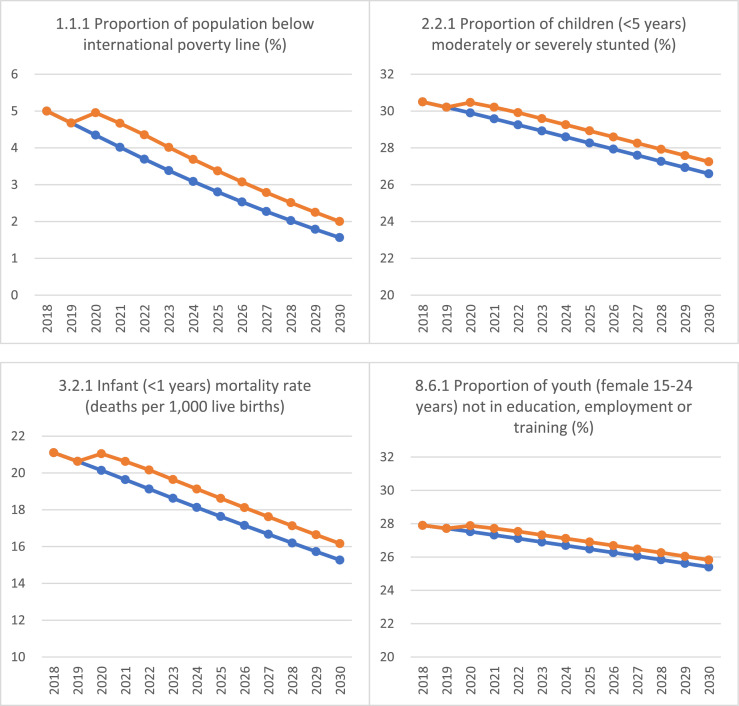
Projection plot for selected SDGs indicators from 2018 to 2030 in Indonesia Note: This figure plots an example of the results with (red line) and without (blue line) COVID-19 projections for selected SDGs indicators in Indonesia. The projected values were calculated from the projected GNI per capita and the estimated elasticity of the SDGs indicator to GNI per capita.

Table 2 provides an example of the projections for four selected SDGs indicators in five ASEAN countries. Fig. 3 depicts the projection plot for Indonesia. As presented in Table 2 and Fig. 3, without the COVID-19 pandemic, the proportion of population below the international poverty line in Indonesia in 2030 is expected to be 1.56%. However, as the pandemic hit the country, the poverty rate increased to 2%. In other words, there is a 0.4% gap that is equivalent to a setback of 1.88 years.

**Table 2.** Selected SDGs Indicators Projection in 2030 in ASEAN-5 Countries

Indicator Code	Indicator Name	Dimensions	Country	2030 Projection			
				Without Covid-19	With Covid-19	Gap (%)	Lag (Year)
1.1.1	Proportion of population below international poverty line (%)		Indonesia	1.564	2.001	0.444	1.878
			Malaysia	0.000	0.000	0.000	0.000
			Philippines	0.000	0.511	0.511	2.000
			Thailand	0.000	0.000	0.000	0.000
			Vietnam	0.000	0.000	0.000	0.000
2.2.1	Proportion of children moderately or severely stunted (%)	<5Y	Indonesia	26.597	27.248	0.887	1.922
			Malaysia	17.259	17.812	0.668	2.177
			Philippines	25.639	26.836	1.610	2.923
			Thailand	6.875	7.481	0.650	2.225
			Vietnam	17.850	18.116	0.324	0.578
3.2.1	Infant mortality rate (deaths per 1,000 live births)	BOTHSEX-<1Y	Indonesia	15.267	16.164	1.058	1.912
			Malaysia	3.676	4.198	0.542	2.117
			Philippines	15.225	16.923	2.002	2.869
			Thailand	3.705	4.442	0.765	2.176
			Vietnam	6.385	6.841	0.487	0.581
8.6.1	Proportion of youth not in education, employment, or training, by sex and age (%)	FEMALE-15-24	Indonesia	25.403	25.826	0.566	1.923
			Malaysia	13.942	14.374	0.502	2.202
			Philippines	22.737	23.507	0.997	2.926
			Thailand	16.871	17.277	0.489	2.234
			Vietnam	7.218	7.385	0.180	0.577

Notes: This table presents an example of the 2030 projections of four SDGs indicator-series-dimensions for five ASEAN countries with and without the impact of the COVID-19 pandemic. It also considers the projection gap and lag. For example, the indicator 2.2.1 “Proportion of children moderately or severely stunted (%)” in Indonesia without the COVID-19 pandemic is projected to be as high as 26.597% in 2030. However, when the COVID-19 pandemic is considered, the prevalence of stunting is projected to be a bit higher at 27.248%. This is equivalent to a 0.887% gap or a 1.922 years setback.

## Concluding remark

This study is related to the study of Komarulzaman et al. [1] and analyzes the impact of the pandemic on achieving the SDGs. It proposes a protocol for creating datasets comprising SDGs indicator projection for 2030, considering the impact of a large economic shock. The protocol is created by first filtering the UNSTAT SDGs indicators for regression analysis to connect the indicators with economic growth. It then resulted in the estimated elasticity of the SDGs indicator to GNI per capita, which, combined with the projected GNI per capita, can be used to calculate the projected value of the SDGs indicator to 2030 with and without the shock. We apply this protocol to estimate the impact of the COVID-19 pandemic on achieving the SDGs indicators in ASEAN-5 countries. The resulting projection dataset can be used to monitor how previous projection trajectories of SDGs indicators are affected by relevant large economic shocks, such as those caused by the COVID-19 pandemic. This information is particularly useful to those in charge of planning, monitoring, and evaluating the SDGs agenda, as well as researchers working on SDG-related issues. Although the protocol is applied to COVID-19 in ASEAN-5 countries, the protocol may apply to other economic shocks and countries.

A limitation of this study is that many SDGs indicators are filtered out, because of a lack of association with economic growth. The majority of such indicators is in the environmental pillar. Future research should aim to fill this gap. We suggest that projections data be published regularly by some prominent institutions (e.g., the IMF or World Bank) and include variables other than economic growth, such as unemployment, exports, or consumption. Certain SDGs indicators may have a stronger association with these variables. Future research may look at these other key variables.

## Declaration of Competing Interest

The authors declare that they have no known competing financial interests or personal relationships that could have appeared to influence the work reported in this paper.

## Data Availability

The SDGs data is available online at UNSTAT website. The resulting data is attached as supplementary material.
